# Evolution of pathogen tolerance and emerging infections: A missing experimental paradigm

**DOI:** 10.7554/eLife.68874

**Published:** 2021-09-21

**Authors:** Srijan Seal, Guha Dharmarajan, Imroze Khan

**Affiliations:** 1 Ashoka University Sonepat India; 2 Savannah River Ecology Laboratory, University of Georgia Aiken United States; Ulm University Medical Center Germany; Pennsylvania State University United States

**Keywords:** pathogen tolerance, inflammation, coevolution, spillover, zoonotic diseases

## Abstract

Researchers worldwide are repeatedly warning us against future zoonotic diseases resulting from humankind’s insurgence into natural ecosystems. The same zoonotic pathogens that cause severe infections in a human host frequently fail to produce any disease outcome in their natural hosts. What precise features of the immune system enable natural reservoirs to carry these pathogens so efficiently? To understand these effects, we highlight the importance of tracing the evolutionary basis of pathogen tolerance in reservoir hosts, while drawing implications from their diverse physiological and life-history traits, and ecological contexts of host-pathogen interactions. Long-term co-evolution might allow reservoir hosts to modulate immunity and evolve tolerance to zoonotic pathogens, increasing their circulation and infectious period. Such processes can also create a genetically diverse pathogen pool by allowing more mutations and genetic exchanges between circulating strains, thereby harboring rare alive-on-arrival variants with extended infectivity to new hosts (i.e., spillover). Finally, we end by underscoring the indispensability of a large multidisciplinary empirical framework to explore the proposed link between evolved tolerance, pathogen prevalence, and spillover in the wild.

## Introduction

The frequent emergence of infectious diseases from wildlife and cross-species spillover has transformed the curiosity of understanding the natural variation in host-pathogen interactions into a pressing need (Bloom et al., 2017; Cunningham et al., 2017). Detailed knowledge of circulating pathogenic strains and heterogeneities in infection outcomes and disease dynamics can shed light on potential future transmission events. Tracking ecological conditions underlying spillover events, where zoonotic pathogens overcome the species barrier (i.e., a hindrance to interspecies transmission) to infect a novel host, can be beneficial for predicting the emergence and spread of pathogens. So, what facilitates such spillover? While we have just begun to understand the patterns and processes underlying emerging infectious diseases (EIDs), earlier surveillance of wild animals that are typically known to harbor zoonotic pathogens has revealed certain intriguing trends (Morse et al., 2012). Hosts that are phylogenetically related tend to share a common pathogen pool, and thus have increased potency to cross-infect each other (Shaw et al., 2020; Wolfe et al., 2007). For example, it is already known that primates harbor a diverse array of pathogens capable of causing severe diseases in humans (Han et al., 2016), including parasites such as *Plasmodium knowlesi* (Sabbatani et al., 2010) or simian immunodeficiency virus (SIV) that underwent host-switching and is the most common ancestor of the human immunodeficiency virus (HIV) (Sharp and Hahn, 2011). Perhaps, in such cases, pathogens do not require major adaptations to spill over into phylogenetically closer organisms due to a relatively lower species barrier (e.g., comparable immune responses and physiological processes), thereby increasing the spillover efficiency. Spatial proximity with reservoir hosts can also lead to increased spillover risk (Davies and Pedersen, 2008). This is exemplified by a diverse array of synanthropic (e.g., brown rat, *Rattus norvegicus*) and domestic (e.g., dog, *Canis lupus familiaris*) species that are known to share more zoonotic pathogens with humans than other animal taxa (Gibb et al., 2020; McFarlane et al., 2012), thereby increasing the risk of host shift. However, in these examples, in addition to spillover via phylogenetic relatedness or spatial proximity, arguably, another important condition can be the circulation of a stable, large, and diverse zoonotic pool in the reservoir species. Indeed, this is corroborated by recent analyses and mathematical models indicating that the number of zoonotic viruses with spillover risk might increase proportionally with the total number (Mollentze and Streicker, 2020) as well as the genetic diversity (Remien and Nuismer, 2020) of viruses maintained inside reservoir animals.

How do reservoir species manage to support the circulation of zoonotic pathogens? The answer perhaps lies in specific ecological, life-history, and physiological features of reservoir hosts that allow both a stable circulation of zoonotic pathogens as well as their continuous shedding into the environmental niche shared with other susceptible species (Gibb et al., 2020). For instance, naive Egyptian fruit bats (*Rousettus aegyptiacus*) can remain infected with the Marburg virus for 7 months after inoculation (Schuh et al., 2017) with little or no clinical disease symptoms. Meanwhile, they can also spread the infection efficiently by contiguous shedding into the ecological space that they share with their conspecifics as well other species, including primates (Rasche et al., 2016). In other less-known reservoirs such as water buffalo (*Bubalus bubalis*), a small number of individuals can shed *Brucella abortus*, the causative agent of brucellosis, persistently at a high level for more than 2 months (Capparelli et al., 2009). Persistent shedding of circulating strains of pathogenic *Escherichia coli* such as O157:H7 from various cattle species has already been reported to cause global outbreaks of gastrointestinal illness in humans (Stein and Katz, 2017). The pertinent question here is, of course, what prevents reservoir animals from eliminating these pathogens via effective immune responses? Although the mechanisms are unclear (Gal-Mor, 2018), these examples perhaps hint at the unique adaption of their immune system. Understanding the ecological contexts and evolution of such interactions between the host immunity vs. pathogens is thus necessary not only to explain the persistence of zoonotic pathogens but also to predict how and when the next spillover may happen.

There is also a growing interest to elucidate the factors driving heterogeneous infection outcomes in reservoir vs. new hosts (VanderWaal and Ezenwa, 2016). For instance, original animal reservoirs harboring pathogens capable of causing severe diseases in other animal hosts, including humans, often do not show disease symptoms themselves (Baker et al., 2013; Guito et al., 2021; Pandrea and Apetrei, 2010). Bats and rodents, which harbor more than 60% of known zoonotic pathogens, are classical examples of such reservoir hosts (Jones et al., 2008) as they are capable of asymptomatically carrying a high diversity of human pathogens, including coronaviruses, henipaviruses, filoviruses, and hantaviruses (reviewed in Subudhi et al., 2019). Recent studies indicate that they are efficient reservoir hosts because their dampened innate immune pathways do not form effective barriers to prevent viral infections, thereby allowing viruses to easily establish stable infection inside the host (Letko et al., 2020). Such reduction in immune responses could also protect hosts from negative consequences of immune activation (Khan et al., 2017a) because, contrary to our expectation, disease symptoms are not always caused by ineffective immune responses, but are often mediated via their overreactivity (Graham et al., 2005). For instance, patients infected with HIV or influenza viruses have high levels of type 1 interferon (IFN) and T-cell activation (Teijaro et al., 2011), which also impose cytotoxicity and immunopathological damages (self-harm) to their own cells and organs (Dybdahl and Storfer, 2003; Hsue et al., 2004; Kaplan et al., 2011). This is possibly also true in the case of the ongoing pandemic caused by severe acute respiratory syndrome coronavirus 2 (SARS-CoV-2, Dec 2019 to present), which has already caused more than 4.1 million deaths within 1.5 years (https://covid19.who.int/). Growing evidence suggests that besides causing severe flu-like symptoms in humans (Harrison et al., 2020), SARS-CoV-2-driven increased morbidity is also associated with a ‘cytokine storm’ comprising surplus release of tumor necrosis factor-α (TNF-α) and IFN-ɣ (Ayres, 2020; Azkur et al., 2020), triggering multiorgan failure and sepsis (Hu et al., 2021). Certainly, the answers to such heterogeneous infection outcomes perhaps lie in – why do different hosts, in the first place, employ distinct immune response strategies against the same pathogen?

**Figure 1.**
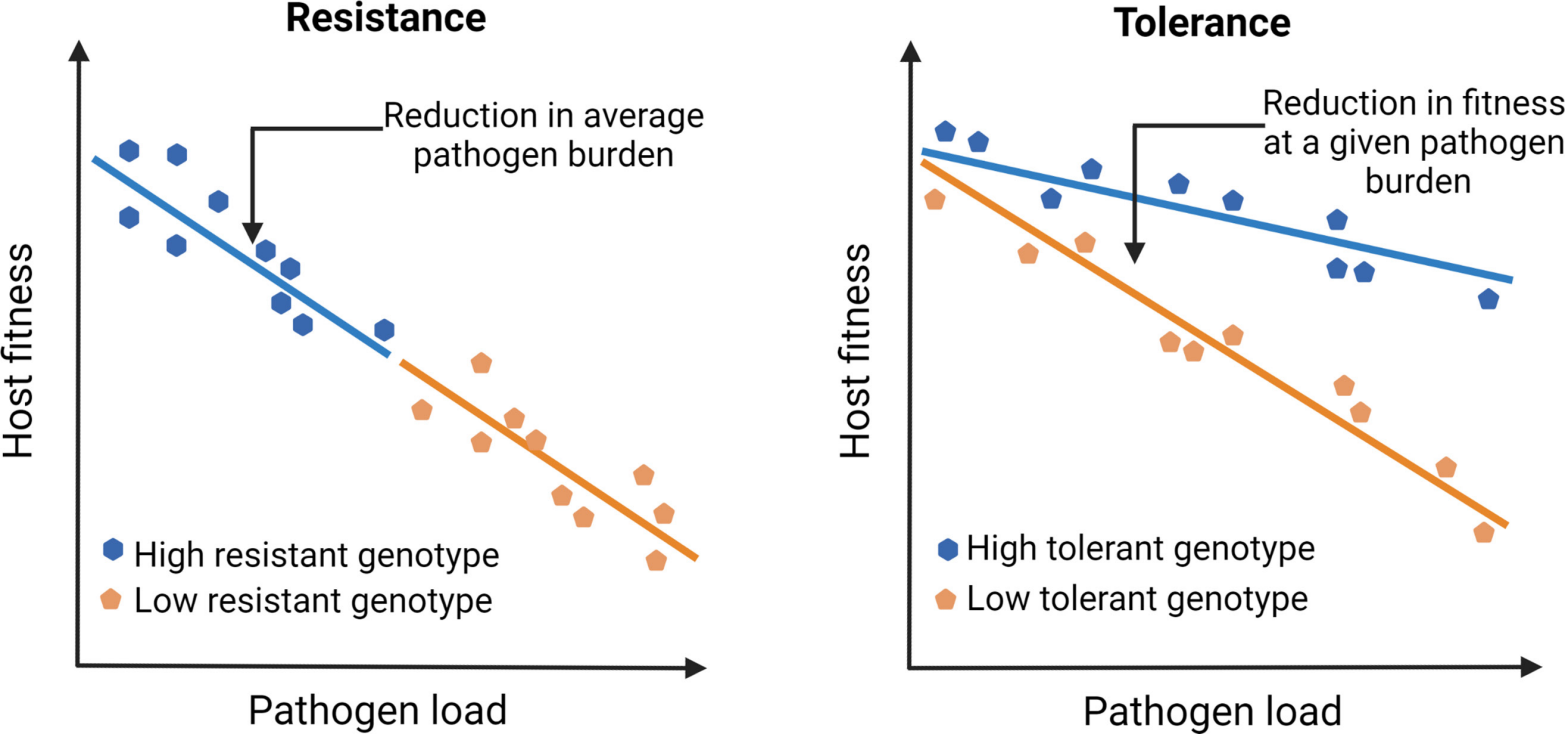
Outlining the difference between resistance vs. tolerance. Defense mechanisms against invading pathogens can either include eliciting immune responses to detect and eliminate pathogens (resistance) or mitigate the fitness costs of infection or immune activation without directly reducing the pathogen load (tolerance). Different genotypes are initially exposed to the same number of pathogens. Figure plotted based on hypothetical data and adapted from Figure 1 of Råberg et al., 2007.

Unfortunately, our understanding of infection and disease has been overtly biased by how we perceive pathogens that infect us. Since pathogens by definition reduce host fitness (e.g., through increased mortality or reduced fecundity), host-pathogen interactions have been traditionally viewed as purely antagonistic. Consequently, studies on pathogen defense have primarily focused on mechanisms that host typically use to resist infections by activating immune responses (Ayres and Schneider, 2012). This bias has led us to ignore mechanisms that facilitate the host’s ability to coexist with pathogens and withstand their negative fitness effects by reducing pathogen- or immune-mediated damage (i.e., tolerance; see Figure 1; McCarville and Ayres, 2018; Råberg et al., 2009; Råberg et al., 2007; Schneider and Ayres, 2008). Such a response to tolerate pathogens and their effects is perhaps a more meaningful strategy from the reservoir host’s perspective (discussed later). Contrary to pathogen resistance, since tolerance mechanisms mitigate fitness costs without directly changing the pathogen burden, they can explain their high abundance and longer persistence required for effective transmission of emerging infections (Mandl et al., 2015; Oliveira et al., 2020). However, despite the proposed link (e.g., high circulation of Marburg virus in bat hosts; Guito et al., 2021) or SIV in simian hosts, (Chakrabarti, 2004) causal connections between tolerance, pathogen circulation, and risks of emerging infections in the natural host-pathogen systems have been rarely analyzed (but see Guito et al., 2021); for example, at an ecological scale, how do host immune strategies and pathogen populations interact to modulate the risk of emerging infections? Indeed, studies of several emerging viral diseases in human cell lines and other laboratory models have been highly successful in shedding light on proximate host defense mechanisms and counter-strategies used by viruses (Legrand et al., 2006). Yet they might not be the best system to understand infections in their natural hosts (Bean et al., 2013) and simulate situations where they can become an emerging infection in the wild (Flies, 2020a).

**Figure 2.**
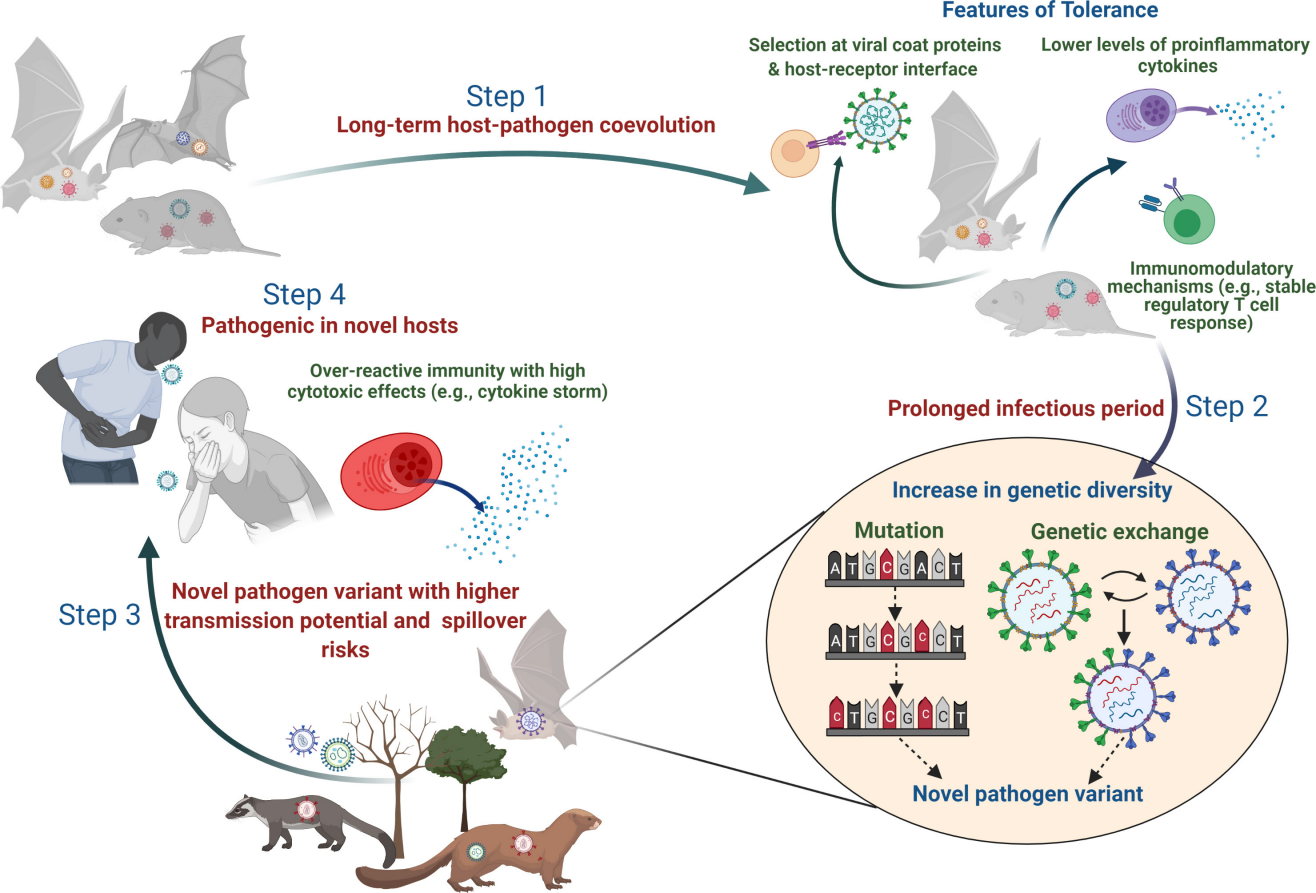
Plausible sequence of events leading to the emergence of zoonotic diseases from tolerant reservoir hosts. Step 1: Long-term coevolution with natural pathogens might lead to specific adaptation of the immune system of reservoir species such that they can tolerate pathogens by reducing their counteractive inflammatory responses or evolving various immunomodulatory responses (e.g., altered activity of regulatory T-cells; Pavlovich et al., 2018; Robertson and Hasenkrug, 2006). Step 2: Tolerant hosts with reduced inflammatory responses can support the circulation of diverse pathogen species and strains. A longer infectious period within tolerant hosts also provides an accurate ecological niche where pathogens can acquire newer mutations over time or undergo genetic exchanges and re-assortments to produce novel variants (Bhat et al., 2021; Domingo-Calap, 2019). Step 3: Although the risk of infecting a new host species (other than the natural reservoir species) might be low, some of these pathogen variants with altered genetic backgrounds might be able to cross the species barrier and infect a new host more effectively (Mandl et al., 2015). Step 4: When novel hosts (e.g., humans or domestic animals) come into contact with these pathogens, they might face severe illness as they are unable to tolerate the impacts of infection or lack mechanisms to reduce the cytotoxic effects of inflammatory responses (e.g., cytokine storm during SARS-CoV-2; Azkur et al., 2020).

In this review, we are primarily addressing how disease tolerance in reservoir species can be intrinsically linked to the maintenance and transmission of pathogens and their spillover. We first discuss why and how tolerance might naturally evolve during long-term association between natural hosts and pathogens as an effective strategy. We then outline the favorable ecological and evolutionary contexts vis-à-vis host-pathogen tolerogenic interactions that may maximize the spillover risk (see Figure 2 for a brief conceptual outline). Besides host immune modulation, we note that tolerance may also evolve because pathogens can adapt to cause less harm to their hosts. Finally, we end by highlighting the importance of a systematic empirical framework to test various hypotheses on disease tolerance and its plausible evolutionary ecological role in emerging infections. With growing evidence of disease tolerance in natural host-pathogen systems, we hope that its detailed understanding might provide new impetus to infectious disease research and pandemic preparedness.

## Relevance of evolving tolerance in natural host-pathogen systems

Immune strategies are not only contingent on how hosts and pathogens interact but also depend on their specific ecological and life-history contexts. Depending on the pathogenicity and infection frequency, the host’s optimal immune response might rapidly change (Khan et al., 2017b; Sorci, 2013). While killing invading pathogens by activating immunity seems to be the most obvious choice for hosts to respond against infection, such resistance mechanisms can themselves lead to negative fitness consequences via immunopathological damages (Khan et al., 2017a; Schneider and Ayres, 2008). Depending on what types of cells and organs are getting damaged, immunopathology can ultimately lead to disruption of normal physiology and impose lifelong pathological consequences (Auten and Davis, 2009; Khan et al., 2017a). Do high costs of such inflammatory responses against pathogens tilt the balance in favor of a tolerance strategy over an evolutionary time scale? Although there are no experiments to detect such dynamic changes in immune strategies, one possibility is that if the activation of the immune response causes proportional damage to both the host and the pathogen, then the host’s ability to invest in self-toxic immune responses might have an upper limit. Beyond this threshold, the host may switch strategy from active resistance to tolerating infections to limit the immunopathological damages. However, tolerance to invading pathogens might also have a threshold, especially when invading pathogens exploit the host resources, and hence, an unlimited number of pathogens is not sustainable. Therefore, optimal use of immune strategies is perhaps fine-tuned by the fitness effects of both immune activities and pathogen statistics (Mayer et al., 2016).

### Tolerance in natural hosts and disease reservoirs

Recent experiments in naturally occurring systems have provided ample evidence for disease tolerance in nature, although exact ecological contexts are widely varied and detailed micro-evolutionary processes remain unclear. For example, the West Nile virus causes significant population declines in most avian hosts, but not in mourning doves (*Zenaida macroura*) where individuals can harbor high viral titers without showing any significant morbidity, suggesting features of infection tolerance (Komar et al., 2003; LaDeau et al., 2007). Hawai‘i ‘Amakihis (*Hemignathus virens*) from low-elevation regions show a reduced rate of weight loss and better physiological condition even during the acute-phase infection with *Plasmodium relictum* than their high-elevation counterparts, indicating their higher tolerance to pathological effects of avian malaria (Atkinson et al., 2013). In the wild-caught field voles (*Microtus agrestis*), mature males can maintain better body condition than immature males while harboring very high macro- and micro-parasite loads, again indicating a higher tolerance (Jackson et al., 2014). Older tadpoles of American toad (*Bufo americanus*) and green frogs (*Rana clamitans*) also show characteristics of relatively higher tolerance to *Echinostoma trivolvis*, a locally abundant trematode species, compared to younger tadpoles (Rohr et al., 2010). Besides showcasing tolerance in natural populations, these examples also highlight how tolerance response in the wild is sensitive to species identity, population history, and their life-history traits.

Molecular information underlying the host’s responses against their natural pathogens further revealed that several key reservoir species have consistently evolved mechanisms to mitigate the immunopathological consequences caused by the over-induction of inflammatory pathways (Letko et al., 2020). A very recent analysis showed that fruit bats (*R. aegyptiacus*)*,* the natural reservoirs for the Marburg virus, lack the induction of several pro-inflammatory genes that are classically implicated in primate filoviral pathogenesis such as CCL8, FAS, and IL6 (Pavlovich et al., 2018). While they have expanded the type I IFN gene family, which is known to initiate an antiviral immune cascade with reduced inflammatory capacity, they also seem to use natural killer (NK) cell receptors with distinct inhibitory signaling components, allowing them to asymptomatically harbor high viral loads (Pavlovich et al., 2018). Also, the PHYIN family of genes and sets of innate immune receptors/sensors capable of activating inflammasome were shown to be absent in two bat species, *Pteropus alecto* and *Myotis davidii* (Ahn et al., 2016). Using different types of RNA viruses such as influenza A virus, Melaka virus, and Middle East respiratory coronavirus, researchers have shown that dampening inflammatory responses enable these bats to tolerate multifarious viral infections (Ahn et al., 2019), avoiding immunopathological damages caused by cytotoxic intermediates (Letko et al., 2020; Subudhi et al., 2019). The reduction in cytotoxic inflammatory responses in bats has been further proposed to have coevolved as a response to minimize DNA damage, caused by free radicals generated during their increased metabolic activity while flying (Irving et al., 2021; Zhang et al., 2013). Such mechanisms also highlight the liaison between bat immunity and key life-history adaptations.

Interestingly, sooty mangabeys (*Cercocebus atys*) and African green monkeys (*Chlorocebus aethiops*) infected with SIV show acute early inflammation, but they also possess regulatory mechanisms to rapidly control such responses; for example, they use anti-inflammatory inhibitory cytokines such as transforming growth factor-β (TGF-β) and IL-10 (Silvestri et al., 2007) to avoid chronic aberrant immune activation and immunopathology (Ansari and Silvestri, 2014; Pandrea et al., 2008; Silvestri et al., 2003). Besides, they are also able to maintain normal rates of peripheral mature CD4^+^ T cell proliferation to compensate for the cytopathic destruction of CD4^+^ T cells post-viral infection (Chahroudi et al., 2012). The role of immunomodulatory molecules is widespread in other reservoir species as well. In rodents, regulatory T cell (Treg) responses suppress inflammation and downregulate cytotoxic T lymphocyte responses that usually eradicate the virus-infected cells, thereby facilitating viral persistence inside hosts (Robertson and Hasenkrug, 2006). For example, hantavirus-infected rodents maintain a steady-state Treg response to allow viral persistence as well as to curb inflammation-induced immunopathology (Schountz and Prescott, 2014). Deer mice (*Peromyscus maniculatus*) infected with Sin Nombre virus (SNV) also upregulate cytokines that correspond to Treg responses, prolonging the viral presence (Ermonval et al., 2016). Norway rats (*R. norvegicus*) infected with Seoul virus (SEOV) not only reduce the pro-inflammatory mediators such as interleukin-6 (IL-6) or TNF-α in their lungs but also increase the expression of regulatory factors TGF-β (overexpressed in bats as well; Silvestri et al., 2007) and FoxP3 to prevent inflammation-related pathology at sites of increased SEOV replication (Easterbrook and Klein, 2008). A growing body of evidence for pathogen tolerance is also coming from arthropod vectors, evolving various mechanisms to efficiently repair the damages caused by pathogens. This in turn allows them to have a normal lifespan while carrying persistent infections. For example, while dengue virus infection in *Aedes aegypti* causes apoptosis in the midgut, mosquito hosts improve the maintenance of midgut homeostasis and tissue integrity via careful regulation of interstitial stem cell (ISC) proliferation, tolerating the effects of viral infection (Oliveira et al., 2020). Another example includes arboviral infection, which usually leads to oxidative stress in insect cells (Joubert et al., 2012), but mosquito vectors can tolerate the infection by upregulating their antioxidant pathways in the midgut (Tchankouo-Nguetcheu et al., 2010; Cappuccio and Maisse, 2020).

Taken together, it appears that reservoir hosts and vectors might have repeatedly evolved either lower inflammatory responses or multifarious compensatory mechanisms to mitigate the negative effects of inflammation. The ability to maintain a balanced immunity and homeostasis during infection might explain their ability to tolerate the circulating pathogens, without showing severe disease symptoms. Yet, a major gap in our understanding is that none of these previous experiments could reveal how these features arose in these animals.

### So, what drives the evolution of tolerance?

Although experimental results are limiting, one of the most compelling results in recent years was obtained from longitudinal sampling of wild Soay sheep (*Ovis aries*) populations performed by Hayward and coworkers (Hayward et al., 2014). They not only showed tolerance in wild sheep populations against their naturally occurring intestinal worms but also provided the conceptual framework for how natural selection might have acted upon tolerance (Hayward et al., 2014). For instance, individuals losing bodyweight more slowly with increasing pathogen burden (i.e., more tolerant, Figure 1) had higher lifetime reproductive success, suggesting a strong positive selection on tolerance. However, the most striking feature of their results was that the observed variations in tolerance were mostly explained by the environmental effects, with very little additive genetic variation left in the population, thereby indicating that tolerance evolved under a strong directional selection. These results conform with existing theoretical models that predicted tolerance to reduce polymorphism, underscoring the importance of directional selection therein (Miller et al., 2005). In other words, as the infection spreads, consistently higher fitness advantage of tolerant hosts than their nontolerant counterparts might reduce the levels of genetic variation and cause rapid fixation of tolerance-related alleles (Miller et al., 2005; Roy and Kirchner, 2000). This is in stark contrast to resistance strategy, which typically reduces pathogen fitness, instigating an evolutionary arms race to select for pathogen traits to overcome the host’s resistance mechanisms (Schneider and Ayres, 2008). However, high costs of immune activation and life-history trade-offs might cause resistance alleles to converge to an intermediate optimum under stabilizing selection (Råberg, 2014). Individuals can also maintain genetic variation for resistance under balancing selection (Råberg, 2014), which might produce highly polymorphic infection outcomes within the population (Lefèvre et al., 2010).

Notably, understanding the evolutionary origin of pathogen tolerance in the wild might require information on the long-term coevolutionary history of natural reservoirs and their pathogens. In most cases, it is quite difficult to validate a causal link between coevolutionary history and micro-evolutionary processes leading to the evolution of tolerance in natural hosts, but a few recent comparative analyses offer some interesting clues. A key experiment with populations of house finches (*Haemorhous mexicanus*) from two locations with a different coevolutionary history of infection by bacterium *Mycoplasma gallisepticum* was particularly helpful here (Adelman et al., 2013). The population from Alabama with a longer history of exposure to *M. gallisepticum* infection showed higher tolerance than the population from Arizona, which was not exposed to the pathogen previously. This is further supported by mechanistic studies, which revealed that the more tolerant Alabama population expressed lower levels of pro-inflammatory cytokine (IL-1β) and higher levels of anti-inflammatory cytokine (IL-10) (Adelman et al., 2013). In another example, natural populations of Asian tiger mosquitoes (*Aedes albopictus*) isolated from regions with longer exposure to heartworm (*Dirofilaria immitis*) also showed higher tolerance compared to populations with little exposure to the parasite (Dharmarajan et al., 2019). These results might have negative implications for human health as tolerant mosquitoes with increased vectorial capacity might catalyze the disease spread (Dharmarajan et al., 2019; Lefèvre et al., 2013). In rodents, phylogenetic analyses have revealed that hantaviruses became associated with ancestral rodents of the family *Muridae* (Plyusnin and Morzunov, 2001). Subsequently, when the ancestral family underwent co-speciation events resulting in different subfamilies such as *Murinae*, *Arvicolinae*, and *Sigmodontinae,* hantaviruses remained associated with them, thereby explaining their continued persistence and asymptomatic state of several rodent species (Plyusnin and Morzunov, 2001; Schountz and Prescott, 2014). Finally, sooty mangabeys and African green monkeys, natural hosts of SIV, also remain healthy and do not develop AIDS (Chahroudi et al., 2012; Wetzel et al., 2017) possibly because of their long coevolutionary history with lentiviruses (dating back to 5–6 million years; Compton and Emerman, 2013), which enables them to prevent the deleterious consequences of SIV infections (Rudensey et al., 1995). Taken together, while these examples unanimously suggest the importance of long-term host-pathogen coevolutionary dynamics in pathogen tolerance, they also indicate that such a response is perhaps unlikely to be true for host species exposed to novel pathogens that they have not coevolved with.

### Implications of land-use changes

In recent decades, the altered trajectory of host-pathogen interactions and coevolutionary dynamics might have more obvious consequences for disease spread from animals to humans, associated with rapid deforestation and land-use changes (Bloomfield et al., 2020; Plowright et al., 2021). For example, landscapes with patches of forests are likely to have increased spatial overlap between wildlife, livestock, and humans. This presents ideal ecological conditions for transmission of zoonotic pathogens from naturally tolerant wildlife hosts, thereby increasing the risk of disease outbreaks in nearby domestic animal or human populations (Hansen et al., 2013; Rulli et al., 2017). In 2019, 14 Chinese workers died in Guyana (South America) while engaged in mining due to infection caused by the fungus *Histoplasma*, rarely found in China but prevalent in America, mostly isolated from soil samples containing decaying bat and bird feces (Wang et al., 2019). This might be an example of how the invasion of humans into the natural ecosystem can expose them to local new infections for which they lack effective immune responses. While it will remain unclear whether the outcome would have been different if Chinese populations had shared evolutionary history with *Histoplasma* in their natural habitat, revealing the causality between coevolution, tolerance, and infection will be a formidable challenge for understanding new EIDs in the wild, warranting closer investigation.

## Role of tolerance in spillover and new infections

Successful spillover to novel host warrants multiple sequential steps (Plowright et al., 2017). Briefly, pathogens should first be released by their reservoir hosts either directly into the environment or a new host through different plausible transmission routes such as consumption, animal bites, or sexual interactions (Webster et al., 2017). Pathogens should then survive until it encounters novel susceptible hosts whom it might infect directly or by undertaking a further round of adaptation to the new host environment (Parrish et al., 2008). Finally, once the pathogen establishes infection in the novel host by evading the immune responses, it then needs to spread effectively in the population (Plowright et al., 2015; Subudhi et al., 2019). At each step of this transmission chain, the duration of the host’s infectivity, population density, and size might dictate the success of the consecutive step (Remien and Nuismer, 2020; Wolfe et al., 2007). However, before all these fine-scale micro-evolutionary downstream processes can begin, potentially an important precondition can be the maintenance of a sufficiently large (Mollentze and Streicker, 2020) and diverse (Remien and Nuismer, 2020) zoonotic pathogen pool with the potential to overcome the species barrier. Although the causal link is absent, large populations of reservoir animals harboring large pathogen populations have been predicted to serve as fertile sources of zoonotic diseases (Han et al., 2016). Also, the role of reduced inflammation and disease tolerance in maintaining such persistent zoonotic pathogen populations in reservoir species has already been implicated (Pavlovich et al., 2018; Martin et al., 2019), but how it can boost transmission and spillover is relatively unclear.

### Tolerance might enhance spillover risk by increasing the infectious period, pathogen burden, and genetic diversity

Physiological mechanisms underlying the tolerance response might be critical in triggering the spillover process (Medzhitov et al., 2012; Henschen and Adelman, 2019). For example, both infectious period and transmission potential can increase if the host tolerates the pathogenic infection by evolving an efficient repair mechanism to counter the damages caused by the pathogen and immune responses (Henschen and Adelman, 2019). The host can generate new cells to replace injured tissues (Medzhitov et al., 2012), as observed in the case of micro-hemorrhages caused by metazoan parasites like *Schistosoma mansoni* or ruptured red blood cells by *Plasmodium* sp. (Allen and Wynn, 2011; Henschen and Adelman, 2019). Such a mechanism can allow pathogens to continuously infect new cells, thereby reducing the selection pressure on them to replicate more effectively (Henschen and Adelman, 2019). Consequently, this whole process might select less virulent pathogens for reservoir hosts (Miller et al., 2006), resulting in a longer infectious period and higher number of circulating pathogens, extended pathogen shedding duration, and increased risk of contacts among infected and susceptible hosts (Adelman and Hawley, 2017; VanderWaal and Ezenwa, 2016). These hypotheses are also consistent with a previous theoretical model, which suggests that tolerance can increase the overall disease burden in host populations, by transmitting the infection to other nontolerant susceptible individuals sharing the same ecological niche (Horns and Hood, 2012). The model further predicts that because of such increased disease burden tolerance is most effective in small and isolated host populations, where the risk of infection transmission to other susceptible individuals can be minimized, suggesting a joint role of demographic features and tolerance on disease spread. A recent study on African straw-colored fruit bats (*Eidolon helvum*) strongly supports this possibility where small isolated populations had a higher abundance of henipaviruses and extended within-host latency (Peel et al., 2018). Although not tested empirically, spatial proximity to these populations can certainly increase the risk of infections to conspecific susceptible individuals as well as spillover to new hosts.

It is also important to note that spillover into a new host is a rare event (Cross et al., 2019) where pathogen abundance alone may not be always sufficient to jump across the species barrier. Although not mutually exclusive, the emergence of novel zoonotic pathogens might also depend on the genetic diversity of the pathogen pool (Wolfe et al., 2007). Pathogen genetic diversity is likely to be greatest within large reservoir populations when they also harbor proportionally large pathogen populations (Remien and Nuismer, 2020). Increased strain diversity might enhance the pathogen’s prospect of jumping across species barrier by harboring the pool of useful mutations to establish infection in a new host (Dennehy et al., 2010; i.e., production of specific rare variants that are inherently more competent to establish cross-species infection; Mandl et al., 2015). Indeed, changes in genetic diversity of the pathogen pool by mutations or genetic exchanges can lead to alterations in the kinetics of viral replication within the natural hosts (Simmonds et al., 2019), modulating the host’s ability to detect antigens and initiate countereffective immune responses (Burmeister et al., 2016; Retel et al., 2019).

### Tolerance vs. pathogen interactions

An interesting situation might arise when hosts harbor multiple pathogen strains thriving together, increasing the level of competitive interactions (Miller et al., 2006). It has been shown that under intense intra-specific competition for available hosts, bacteriophage φ6 that normally infects *Pseudomonas syringae* can also rapidly evolve to infect other novel bacterial hosts such as *Pseudomonas atrofaciens* and *Pseudomonas glycinea* (Bono et al., 2013). While this provides a clear example where the ability to infect new hosts arose as a function of intra-specific competitive interactions, it might be relevant for increased disease transmission and spillover as well, provided the probability of such interactions between zoonotic pathogen strains intensifies inside reservoir hosts. Extended infectious period, higher abundance, and relaxed selection within naturally tolerant hosts can certainly provide the appropriate stage for pathogens to acquire mutations to evolve into a new strain or exchange genetic material between various strains (Domingo-Calap, 2019). These are perhaps more likely for pathogens with multi-segmented genomes such as the influenza virus, where rapid viral replication can increase diversity by allowing the recombination of different genomic segments (McDonald et al., 2016). Revealing the plausible ecological contexts that increase the chances of reassortment (e.g., presence of co-infecting strains; Tao et al., 2015) might be crucial to tracing how novel genome combinations can arise to create influenza subtypes with expanded host range and novel antigenic properties (Bhat et al., 2021; also see Cecilia, 2014 for recombined dengue virus genotypes).

Another plausible example is the gene loss and adaptations during interspecies transmission of SIVcpz, a strain of SIV that naturally infects chimpanzees. Later analyses revealed SIVcpz as a recombinant between two SIV lineages from old-world monkeys with a uniquely reconstructed vif gene (Bailes et al., 2003; Etienne et al., 2013). Although it is unclear where and how such genetic modification took place, this enabled the recombinant virus to antagonize hominid antiviral protein *APOBEC3s* more efficiently, contributing to the origin of the HIV-1 pandemic in humans (Etienne et al., 2013). Most recently, the phylogenetic network approach has revealed that even the VOC202012/01 variant of SARS-CoV-2, which was first reported in the UK in 2020, might have originated through recombination of preexisting virus strains before rapidly spreading into many other countries (Xie et al., 2021). Although direct evidence is lacking, the role of suitable human hosts tolerating the coexistence of multiple strains cannot be ignored (Martin et al., 2011; Simon-Loriere and Holmes, 2011). This is partly corroborated by recent evidence of SARS-CoV-2 evolution in immunocompromised patients who could maintain high viral loads over prolonged periods (reviewed in Day et al., 2020), thereby allowing more opportunities for viral replication, mutations, and potential recombination events.

In contrast, recombination between unrelated groups of viruses is rare, but such situations can also arise, at least ecologically, if they coexist within a tolerant host, serving as a unique niche for them to stay together for long, interchange genomic sequences and undergo recombination to create viral strains with emergent properties. For example, both yellow fever virus (YFV; flavivirus) and SIV (retrovirus) might persist together in their natural hosts sooty mangabeys (Woodall, 1968), which show tolerance to these viruses by significantly reducing the IFN-α level (Mandl et al., 2011). Mosquito host *A. aegypti* is also known to tolerate both dengue (flavivirus) and chikungunya (alphavirus) virus (CHIKV) (Kaur et al., 2018). Although genetic exchanges between such distinct virus lineages might appear far-fetched at present due to a lack of empirical support, scant evidence exists from some environmental isolates (Diemer and Stedman, 2012). For example, viral metagenomic sequences derived from a hot, acidic lake in Lassen Volcanic National Park (USA) have revealed a single-stranded DNA virus encoding a major capsid protein, which is similar to those found only in single-stranded RNA viruses, suggesting a recombinant viral genome (Diemer and Stedman, 2012). This is puzzling because mechanisms for interviral RNA-DNA recombination are unknown (Stedman, 2015). Also, indirect support for the possibility of genetic exchanges between cohabiting distinct viral pathogens might come from a recently identified novel coronavirus (labeled as Ro-BatCoV GCCDC1) found in *R. leschenaultia*, which carried a functional p10 gene (involved in the formation of cell syncytia) possibly derived from a bat-isolated orthoreovirus (Huang et al., 2016). In this example, the putative inter-family heterologous recombination event between a single-stranded RNA virus (i.e., ancestral beta-coronavirus) and a double-stranded segmented RNA virus (i.e., orthoreovirus) hints at possibilities of how specific genetic events might trigger the formation of recombinant viruses in nature with potentially altered transmission potential (Huang et al., 2016). Another example is the novel bandicoot papillomatosis carcinomatosis virus type 1 (BPCV1), isolated from western barred bandicoots (*Perameles bougainville*), which exhibited genomic properties of both the Papillomaviridae and the Polyomaviridae family of viruses (Woolford et al., 2007). These observations (and perhaps many more that await discovery in future) indicate that genetic exchanges between diverse groups of pathogens are indeed possible in natural conditions and such possibilities might increase proportionally with the time spent together inside a tolerant host. For example, longitudinal observation of one population of *Rousettus leschenaultii* bats for 2 years found recombinants of RdRp (RNA-dependent RNA polymerase) and p10 genes in Ro-BatCoV GCCDC1 within as early as 5 months since the initial surveillance began (Obameso et al., 2017).

### Evolution of immune evasion strategies by pathogens

Pathogens, especially viruses, can evolve much faster than their hosts, presenting numerous mechanisms to avoid immune sensing (Silvestri, 2009). SIV, for example, can rapidly produce variants that can escape cytotoxic T lymphocytes of their natural host sooty mangabeys (Kaur et al., 2001). A certain allelic variant of the Nef gene product from SIV downregulates the CD3-T-cell receptor complex from infected CD4+ T cells, suggesting the ability to block the counteractive immune responses and maintain the viral persistence (Schindler et al., 2006). This perhaps also exemplifies how pathogens might adapt to bypass host immunity, promoting a tolerance-like response to avoid harmful effects of immune activation. However, such function was lost during viral evolution in the lineage that ultimately gave rise to HIV-1. Most recently, a novel variant of SARS-Cov2 (B.1.427/B.1.429), isolated from California (USA), was also found to harbor spike glycoprotein mutation that could reduce the neutralization effectivity of the Wuhan-1 isolate-based mRNA vaccine (McCallum et al., 2021), suggesting the possibility of new mutations aiding rapid evolution of the virus against vaccine-elicited antibodies (also see SARS-CoV-2 B.1.617.2 (Delta) variant identified in the state of Maharashtra, India, against BNT162b and ChAdOx-1 vaccines; Kemp et al., 2021).

Conversely, host-pathogen tolerogenic interactions over an evolutionary timescale might also lead to progressive loss of immune evasion mechanisms in the pathogen, potentially reducing their infectivity to future hosts. Perhaps, one of the best-documented examples includes Myxoma virus (MYXV), which is highly pathogenic to European rabbits, with a case fatality rate close to 100% (Peng et al., 2016). The same virus lost its virulence by 50–70% after being introduced in Australia to counter their invasive rabbit populations. During this coevolution, the Australian isolate of MYXV suffered a loss of function mutation in their protein M156, which is critically required to counter host antiviral protein kinase R (Peng et al., 2016). In contrast, SIV is known to retain its infectivity across species even after a long-term transgenerational association with experimentally inoculated monkey hosts, as noted in the case of cross-infection from laboratory macaques to humans (Khabbaz et al., 1994). More studies are perhaps required to test these diverse pathogen-specific outcomes vis-a-vis zoonotic transmission.

### Supporting evidence for tolerance and pathogen prevalence from vaccination studies

Finally, recent vaccination studies in poultry birds can also offer some important clues on how tolerance can in principle influence the pathogen persistence and diversity. This is particularly true for vaccines that operate by reducing the disease symptoms (i.e., leaky vaccines), rather than preventing the infection, pathogen replication, and transmission (Gandon et al., 2001; Read et al., 2015). Infection outcomes in these vaccinated hosts largely resemble several features of tolerance where pathogens do not cause disease despite an extended infectious period (Mackinnon et al., 2008), but become progressively more virulent to other nonvaccinated hosts (compare with Horns and Hood, 2012 model). For example, more virulent strains of Marek’s disease virus appeared, persisted, and were transmitted among chickens when they were vaccinated (Read et al., 2015). Here, the ability to withstand infection via leaky vaccines perhaps provided the ideal ecological conditions that facilitated modified viral strains to emerge, persist, circulate, and transmit effectively, which otherwise would have been lethal for the chicken host to carry.

Another example is live vaccination with attenuated strains of porcine reproductive and respiratory syndrome virus (PRRSV), which prevents the development of disease symptoms in pigs but does not protect them against contracting the infection (Nan et al., 2017). In fact, the application of live vaccines might result in PRRSV variants that cause clinical severity and elevated viremia in the naïve inoculated pigs, suggesting a reversal of virulence level (Jiang et al., 2015; Liu et al., 2018). Although mechanisms are unknown (Zhou et al., 2021), tolerance after vaccination could facilitate genetic diversification of the circulating viruses, enabling them to evolve newer immune evasion strategies and revive virulence. Indeed, a recent study on chicken vaccinated against infectious bronchitis virus (IBV) has detected selection pressure, resulting in the diversification of viral coat proteins (Franzo et al., 2019). This provides a plausible evolutionary mechanism of producing newer vaccine escape variants as well as variants with wider infectivity to new hosts (Franzo et al., 2019). Future studies should thus compare and analyze these different vaccination contexts to verify whether they create likely niches for persistent pathogens and genetic alterations to create new emerging variants – some of them might just be competent enough to cause spillover by infecting new hosts more successfully. We also speculate that underlying mechanisms might be different from that of tolerance achieved via reducing host immune responses, selecting for either loss of immune evasion strategies or lower infectivity of pathogens (discussed above). However, there are no experiments to test whether and how these potentially different tolerance mechanisms might produce contrasting effects on pathogen evolution and emergence.

## An integrated immune-centric experimental paradigm

**Figure 3.**
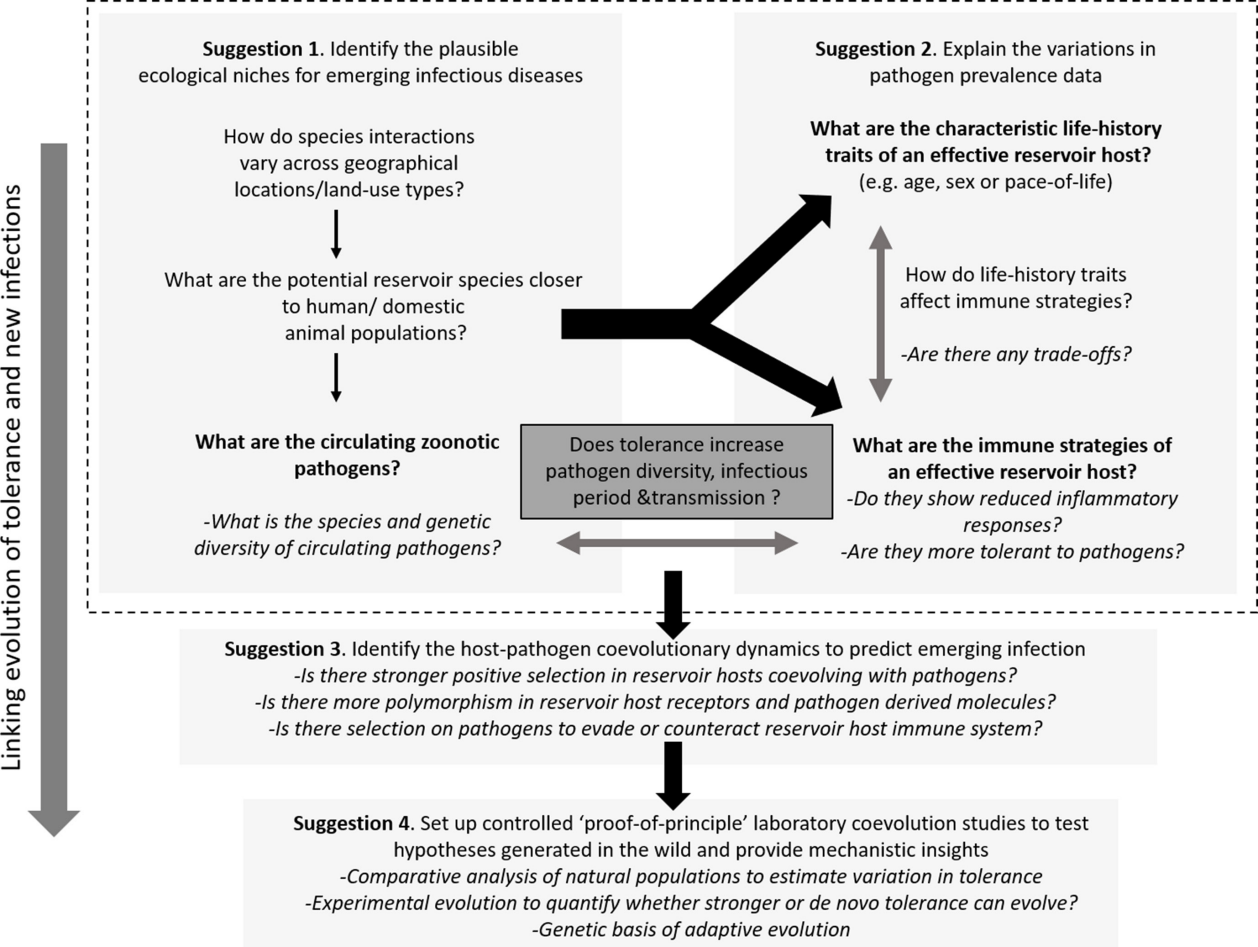
Outline of the proposed experimental paradigm, linking host ecology, tolerance and immune responses, and life-history to understand the natural contexts of emerging infections. While performing the traditional disease surveillance program, information on ecology, life-history, and immune strategies of potential reservoirs can be gathered in parallel to explain why and how zoonotic pathogens are distributed in the wild (Suggestions 1 and 2). Once potential reservoir hosts and zoonotic pathogens are identified, their associations can be tested for signatures of coevolution and compared with overlapping populations of novel hosts (e.g., humans or domestic animals) to analyze the observed polymorphisms of key molecules involved in reservoir host-zoonotic pathogen interactions, divergence in infection outcomes, or immune evasion strategies of pathogens (Suggestion 3). Finally, controlled laboratory experiments can be performed to test causal links between host-pathogen coevolution, pathogen tolerance, and prevalence (Suggestion 4).

Host immune strategies, ecology, and pathogen prevalence all play instrumental roles in facilitating spillover, but studying them in isolation is far from ideal given the complex interactions that are involved therein. Costly immune responses might evolve to act at suboptimal levels in the wild due to constraints from available resources and physiological states (Viney and Riley, 2017). Although large-scale research focusing on model host-pathogen interactions has mostly studied molecular aspects, there is a growing consensus that in the wild, host ecology, life-history, and physiological constraints are important mediators of optimal immune strategies, infection risk, and myriad infection outcomes (Graham, 2021; Restif and Graham, 2015). An integrated approach is thus needed where they should be jointly studied to explain the patterns and processes of pathogen prevalence and infection outcomes in the wild. Below, we suggest a few interrelated research foci that can be combined with traditional disease surveillance programs, aiding biological risk assessment of future EIDs (see Figure 3 for a brief outline of the suggested experimental paradigm).

### Suggestion 1: identify the plausible ecological niches for emerging infections

Our understanding of emerging diseases from natural reservoirs has increased substantially over the past two decades (White and Razgour, 2020), but unfortunately, this knowledge is limited to a handful of species under scrutiny from specific geographic locations. For example, rodents and bats are of special interest from a human disease perspective since they harbor about 60 and 30% of known zoonotic viruses, respectively (Johnson et al., 2020). However, it is often overlooked that they also commonly utilize landscapes frequently occupied by other species, including humans and domestic animals (Morand et al., 2014), increasing the possibility of exchanging microbes at multiple interfaces of species interactions. They might not always cause disease outbreaks, with most of them being benign transfers, but they can help to estimate the risk of the background spillover rate among hosts of different taxa (Flores et al., 2017; Gao et al., 2016).

Transmission dynamics might also be contingent on intermediate hosts and vector populations (Plowright et al., 2017). Understanding pathogen persistence and release from intermediate hosts can lead to unearthing important bottleneck events during the emergence of novel infectious diseases (Cui et al., 2017). Hence, in addition to traditional practices of selectively obtaining data from only a very few overtly represented reservoir species from any location (Watsa, 2020), future efforts can be directed towards continuous monitoring of pathogen abundance and strain diversity across different interacting species occupying the same niche, including potential reservoirs, intermediate and human hosts. Further, it is important to collect such data simultaneously from various landscapes with altered species interactions and community composition because each location provides unique ecological niche catering to diverse host-pathogen interactions. More information across different locations can eventually motivate powerful comparative analyses to uncover novel associations between new host species (or populations) and future zoonotic routes.

Long-term tracking of pathogens and disease with altered species interactions is perhaps most relevant for rapid land-use changes in recent decades (Guo et al., 2019) – deforestation and the resulting loss of biodiversity have already been identified as one of the major driving forces influencing the risk of disease spread from animals to humans (Daszak et al., 2000; Gibb et al., 2020; Patz et al., 2008). Some of the ecological mechanisms influencing disease transmission in anthropogenically modified habitats are certainly the changes in the niche of the interacting species (host/vector/pathogen), their altered behavior, distribution in space, and animal movement patterns (Gottdenker et al., 2014). The relative importance of one or more of these mechanisms in explaining the response to land-use changes is likely to vary across regions. For instance, South Asia has undergone large-scale land conversions at alarming rates, losing approximately 30% of its forest land (Sudhakar Reddy et al., 2018) and, hence, can be the hotspot for EIDs (Coker et al., 2011). We thus strongly recommend a long-term disease surveillance program where multiple such regions should be first identified and then jointly analyzed to understand whether and how altered species interactions are responsible for pathogen abundance and occurrence in different animal hosts across ecosystems. This should be closely followed by tracking how they in turn influence the pathogen communities (with zoonotic potential) found in overlapping human populations.

We note that PREDICT, an epidemiological research program funded by the United States Agency for International Development (USAID), was operational until recently to identify broad patterns of emerging pathogens with pandemic potential in geographical regions that are disease hotspots such as the Republic of Congo, China, Egypt, India, and Malaysia (Karesh, 2011). However, after a decade from its inception, the PREDICT program ended a few weeks before the SARS-CoV-2 pandemic began. In the spirit of PREDICT, there are now several other global surveillance projects that aim to identify novel pathogens before they emerge in human populations. The Global Virome project focuses on the discovery of zoonotic viruses in the hope to prevent the next pandemic (https://www.globalviromeproject.org; Carroll et al., 2018). Similarly, two other programs focus on cataloguing the diversity of life on earth (including pathogens and parasites). These include BIOSCAN (https://ibol.org/programs/bioscan), the extension of the International Barcode of Life Program (Hobern, 2021), and the Earth BioGenome Project (https://www.earthbiogenome.org; Lewin et al., 2018). Programs, like the ones cited above, have generated a lot of basic knowledge on mammalian pathogens, especially viruses, and have aided the recent pandemic effort by understanding potential spillover pathways, as well as the ability to rapidly isolate and classify SARS-CoV-2 (Carlson, 2020). However, it is critical to remember that spillover events that spark pandemics are inherently stochastic, and there continues to be doubt on the direct abilities of programs, like PREDICT, to prevent future pandemics (Holmes et al., 2018). Thus, funding of these programs should not detract from the need for increased funding to monitor at-risk human populations, such as those living in areas of high spillover risk (Holmes et al., 2018). Additionally, there is an urgent need to support and expand networks that aim to rapidly disseminate epidemiological information such as the WHO’s Global Outbreak Alert and Response Network (GOARN), Global Initiative on Sharing All Influenza Data (GISAID), and preprint servers such as Virological (http://virological.org). Finally, proactive programs aimed at pandemic prevention also critically need more spatial and temporal information describing key changes in ecological communities (e.g., biotic homogenization) and environmental parameters (e.g., global climate change) to better understand why circulation and transmission risk of zoonotic pathogens might vary across ecosystems. A recent web-based application ‘SpillOver’ is particularly useful to gain some of these insights (Grange et al., 2021). In addition to information on viruses (e.g., virulence, breadth of viral infectivity), hosts (e.g., genetic relatedness of hosts to humans, the severity of disease in humans), and the environment (e.g., deforestation, land use), the application also considers other ancillary factors such as frequency and intimacy of human interactions with wild and domestic animals to calculate the spillover risk of 887 wildlife viruses and assess their pandemic potential. We hope that more data on host-pathogen communities and their myriad interactions and effects at the backdrop of various human interventions will further improve its location-specific predictive power.

### Suggestion 2: explain the observed variations in pathogen prevalence data

An integrated program to catalog pathogens across species, populations, and locations will prepare a unique stage to subsequently ask more mechanistic questions; for example, explaining the macro-scale structural variations, using diverse metrics of host immunological, ecological, and physiological parameters. However, multiple challenges need to be overcome to conduct any meaningful analyses. Below, we describe the indispensability of accepting the challenges and testing the natural variation in immune strategies and their complex interplay with life-history to explain EID prevalence and emergence.

#### A. Role of immunity and tolerance

While the role of host immune responses in shaping heterogeneous infection outcomes (Duneau et al., 2017) and pathogen evolution (Retel et al., 2019) is unquestionable, their importance in driving naturally occurring variations in pathogen prevalence should gain more attention. Tracing the link between variations in inflammatory responses, tolerance, pathogen abundance, and diversity can provide insightful evidence about how the infection outcomes and their downstream effects on pathogen transmission vary across reservoir species populations. However, estimating zoonotic pathogen load in wild reservoirs and linking them to changes in their fitness proxies (i.e., tolerance; changes in the slope of fitness-by-pathogen load; Ayres and Schneider, 2012) can be notoriously difficult because of poor field understanding of their biology and lack of controlled experimental paradigm. Besides, it is often not feasible to use similar fitness parameters to measure tolerance across species and pathogens due to differences in the mode of pathogenesis and physiological processes involved. Conversely, tolerance is easier to estimate where pathogen-specific impacts on measurable host fitness parameters are known. For example, a recent study standardized skin lesion prevention efficacy as an important fitness proxy to estimate tolerance in salamander species to a pathogen *Batrachochytrium salamandrivorans* that usually infects amphibian skins (Wilber et al., 2021). In contrast, another study used the extent of fin damage caused by ectoparasite *Tracheliastes polycolpus* (which feeds specifically on and destroys fins) as a proxy in a wild population of cyprinid freshwater fish (*Leuciscus burdigalensis*) (Mazé-Guilmo et al., 2014). In Atlantic salmon (*Salmo salar*), tolerance was quantified by assessing eye cataract formation as a degree of pathology against increasing burden of eye fluke *Diplostomum pseudospathaceum* (Klemme et al., 2020). All these examples suggest that we need to design long-term studies to first understand the basic life history of key reservoir species in the wild to standardize fitness measurements and observe their response to pathogens of significant zoonotic interests (e.g., counting number of circulating hemocytes, antibody titers). This will enable us to understand the actual ecological role of zoonotic infections and disease manifestation in wild host populations. Moreover, long-term studies are also important to reveal how selection acts on the host immune system against pathogens of interest. For instance, Sparks and coworkers collected data for 26 years from wild Soay sheep (*Ovis aries*) populations to distinguish how natural selection acts separately on three functionally distinct isotypes of antibodies (IgA, IgE, and IgG) against a prevalent nematode parasite *Teladorsagia circumcincta* (Sparks et al., 2018). Future studies might consider comparable frameworks to reveal the mechanistic basis of how immune system might evolve with pathogen burden and is linked to fitness effects in reservoir hosts against zoonotic pathogens.

A few earlier studies have successfully looked into tolerance by estimating fitness traits such as body mass, standing pathogen load, lifespan, and number of offspring produced per year in rodent populations (Jackson et al., 2014; Rohfritsch et al., 2018; Schneider, 2011), which can be replicated in future as well. A recent study with natural populations of *Anolis sagrei* lizards also used body condition, locomotor performance, and survival to the end of the breeding season as a function of infection with *Plasmodium* parasites (Bonneaud et al., 2017). However, rodents can be used as a more relevant model species to link tolerance with emerging infections as they are one of the largest disease reservoirs (Gravinatti et al., 2020), ubiquitously found in all ecosystems. Also, the immune system of several highly abundant rodent species such as *Rattus rattus* or *Mus musculus* is very well-characterized (Abolins et al., 2017; Viney et al., 2015), providing the opportunity to correlate known immune parameters against zoonotic pathogens with measurable fitness traits. Future studies can also design these assays in various ecosystems based on previous rodent experiments, where both cross-sectional destructive sampling to obtain precise measurements as well as longitudinal sampling using the capture-recapture method were implemented to provide stronger causal inferences (Jackson et al., 2014).

Obtaining reliable molecular biomarkers of immunity in wild reservoirs is also important to provide direct evidence for how inflammatory responses might vary across spatial and temporal scales and allow some hosts to tolerate the pathogen, while others cannot (Burgan et al., 2018; Jackson et al., 2014). Indeed, a major challenge is the lack of reagents such as monoclonal antibodies for most wild species, but an increase in the number of fully sequenced genomes and de novo transcriptome assemblages of different reservoirs species in the ecological community can overcome these limitations. This information can enable us to compare the immune-related transcripts and gene expression patterns to produce cross-reactive recombinant proteins for protein-based assays across taxa (Flies et al., 2020b). Indeed, recent efforts have also been successful to sequence whole genomes of different bat species (Jebb et al., 2020). The Bat1k genome consortium aims to sequence and annotate chromosome-level genome assemblies of all living bat species to probe genetic mechanisms underlying their unique adaptations to zoonotic viruses (Teeling et al., 2018). Additionally, developing standardized sets of reagents for rapid serological assays of immunoglobulins and key cell types such as resident memory T cells that can react across species can also be extremely helpful to track how species interactions within an ecological niche can influence the possibility of shared zoonotic pathogen pools (Flies et al., 2020b).

#### B. Complex interplay with life history

Host immune strategies and disease tolerance might explain pathogen abundance and strain diversity, but they are unlikely to work in isolation without a whole organismal and physiological perspective. This is primarily because immune strategies are contingent on diverse life-history parameters such as age, sex, reproductive status, or body condition (Nystrand and Dowling, 2020; Poirier, 2019; Smith et al., 2019). A previous meta-analysis by Han and colleagues (Han et al., 2016) has identified a diverse array of life-history traits such as gestation length, longevity, group size, mating system, offspring per year, and age of sexual maturity that makes certain species ideal as zoonotic reservoir hosts. However, these patterns make more sense if analyzed in terms of how hosts at a particular life-history condition could maintain pathogens by altering their so-called combative and counteractive immune strategies (Valenzuela-Sánchez et al., 2021; see Table 1 for a few examples highlighting life-history traits and their proposed role in immunity and tolerance).

**Table 1.** Plausible effects of different life-history traits on immune responses and tolerance. Although resource availability and nutrition are not life-history traits, we listed them as they impact body condition and fitness (Huang et al., 2013). ‘↑’ denotes increase in tolerance while ‘↓’ denotes decrease in tolerance.

Traits	Plausible effects on infection and immunity	Reference	Predicted role in tolerance
Reproduction	Higher investment in reproductive output trades-off with immune responses	Short et al., 2012	Higher reproduction↑
Mating effort	Increased mating activity leads to immune suppression	Rolff and Siva-Jothy, 2002	Increased mating effort↑
Pace of life	Fast pace of life reduces investment in immunity and allocate more resources to early-sexual maturity and -reproductive output	Previtali et al., 2012	Animals with fast pace of life↑
Sex	*Option 1*: Males invest less in immunity due to high intra-sexual competition for females and variation in reproductive success	Bateman, 1948; Zuk and Stoehr, 2002	Males↑*
	*Option 2***:** Males disperse more and hence, are exposed to increasing number of (diverse) pathogens	Streicker et al., 2016	Males↑
	*Option 3*: Organ-specific localization and impacts of pathogens across sexes; e.g., pathogens that could infect and colonize only ovary might show female-specific pathology	Brandt and Schneider, 2007	Males↑
Age	Younger individuals maximize their reproduction by minimizing investment in immune activation and pathogen clearance, thereby avoiding fitness-reducing immunopathological consequences	Khan et al., 2017a; Medzhitov et al., 2012	Younger individuals↑
Starvation/ availability of resource	*Option 1*: Organisms invest less in immune defenses when deprived of resources	Stucki et al., 2019	Starving individuals↑
	*Option 2*: Well-fed individuals can withstand the effects of pathogens without clearing them because of their better body condition	Knutie et al., 2016	Well-fed individuals↑
Nutrition	*Option 1***:** Access to a proteinaceous diet might boost the immune system and pathogen clearance ability	Povey et al., 2009	Higher protein content↓
	*Option 2***:** Access to a proteinaceous diet might allow hosts to compensate for the costs of harboring pathogens	Knutie et al., 2017	Higher protein content↑

* Also see contrasting examples in Jarefors et al., 2006 or Cousineau and Alizon, 2014; females invest more in anti-inflammatory responses which might increase their tolerance.

Males are more likely to harbor a greater diversity of pathogens compared to females due to their increased propensity to disperse, exposing them to encounter more pathogens (Streicker et al., 2016). Also, host systems that are sexually dimorphic in immunity and infection outcomes can provide the pathogen with two selectively distinct environments (Gipson and Hall, 2016; Khan and Prasad, 2011), imposing far-reaching impacts on disease transmission, especially in populations with skewed sex ratios. Life-history traits such as lifespan, sexual maturity, and reproductive output that make certain species ideal for natural reservoirs (Han et al., 2016) can perhaps be mediated via resource allocation trade-offs (Schwenke et al., 2016), where limiting the activation of costly immune responses might promote other fitness traits and favor pathogen tolerance. For example, several host species like rodents that thrive in human-dominated landscapes usually have a fast pace of life, reducing investment in immunity and thereby harboring more pathogens at any given timepoint (Gibb et al., 2020) – early maturity and high early-reproductive output (e.g., increased reproduction at a young age; see Medzhitov et al., 2012) can trade-off with immune responses allowing rodents to become competent natural reservoirs for zoonotic pathogens (Ostfeld et al., 2014). In nature, frequently encountered stressful environments such as poor nutrition can also have severe impacts on immune investment and pathogen tolerance (Wang et al., 2016). For example, burying beetle (*Nicrophorus vespilloides*) feeding on a low protein diet showed increased tolerance to *Photorhabdus luminescens* (Miller and Cotter, 2018). Another study on Cuban tree frog (*Osteopilus septentrionalis*), however, found increased tolerance to skin penetrating nematode *Aplectana* sp. when maintained on proteinaceous insect diets (Knutie et al., 2017), suggesting that impacts of nutrition on tolerance are perhaps host- and pathogen-specific. Nonetheless, considering these multifaceted implications of host physiology and various life-history traits in immunity and disease tolerance, it is imperative to analyze the role of these parameters in explaining pathogen prevalence data collected during disease surveillance. Different life-history traits can interact closely to drive the level of and pathogen prevalence. For instance, in addition to dispersal (discussed above; Streicker et al., 2016), higher pathogen burden in males can be explained by their lower investment in immunity as they typically experience stronger intra-sexual competition and a greater variance in reproductive success (Bateman, 1948; Zuk and Stoehr, 2002). As implicated above, even age, sexual maturity, pace-of-life, or nutritional status might manifest their effects on immunity and tolerance by altering reproductive investments. Sexually dimorphic immune investment, causing sex-specific divergence in tolerance and pathogen prevalence, may evolve as a function of intersexual resource competition (Li and Kokko, 2019) or mating strategies (Bagchi et al., 2021) We certainly need future studies to explore these intimate interactions and pinpoint complex life-history and physiological features of reservoirs that can be attributed to their disease-carrying ability.

### Suggestion 3: identify the host-pathogen coevolutionary dynamics to predict emerging infections

Analyzing changes in genes involved in host-pathogen interactions can generate crucial insights about their association over an evolutionary timescale (Woolhouse et al., 2002). For instance, virulence genes involved in continuous host-pathogen arms race tend to display positive selection (dN/dS > 1) in the codons that are involved in the interaction sites between the virus and host cell receptor (Daugherty and Malik, 2012; Meyerson and Sawyer, 2011). Indeed, host cell receptors for viruses like HIV (cluster of differentiation 4), filovirus (Niemann-Pick C1), and several coronaviruses (angiotensin-converting enzyme 2 ) have been shown to undergone positive selection across different mammalian orders (Pontremoli et al., 2016; Wang et al., 2020). SIV envelope protein binding domain of CD4 receptor (D1 domain) in many African primate species has undergone diversification in the coding sequence (Zhang et al., 2008). The amino acid replacements in the D1 domain can prevent viral envelope glycoprotein (Env)-CD4-mediated cell entry in multiple African primate species, protecting them from SIV infection (Zhang et al., 2008). A recent study has further revealed that diversification of CD4 receptor, with as many as 11 coding variants in Moustached monkey *Cercopithecus cephus*, might be under balancing selection as an outcome of the long-term coevolutionary arms-race with primate lentiviruses (Russell et al., 2021). Quantifying selection pressures acting at various host receptor-pathogen interfaces by calculating respective dN/dS ratios (Yang and Bielawski, 2000) can thus help us in unearthing evidence of the evolutionary history of exposure; for example, (1) high or low degree of filovirus exposure to natural reservoir bat vs. novel human hosts respectively; or (2) genomic signatures left by pathogenic primate lentiviruses in susceptible primate species vs. other long-term SIV-infected species such as sooty mangabeys evolving specific mechanisms to avoid the disease progression (Russell et al., 2021). The consequences of long-term positive selection on pathogens might also transcend into evolved variants with new antigenic properties and possible expansion of the host range (Bedi et al., 2013). Indeed, in the case of SARS outbreak in 2002, selection on the spike gene of SARS-CoV was positively correlated with its spillover from palm civets to humans (Chinese SARS Molecular Epidemiology Consortium, 2004). The binding affinity of the virus spike protein towards human ACE2 changed from low to high due to mutations in two critical amino acids, turning it into a pandemic strain (Chinese SARS Molecular Epidemiology Consortium, 2004).

Levels of pathogen sequence divergence can accelerate more with increased polymorphism of host receptors (Gupta et al., 2009; Meyerson and Sawyer, 2011; Warren et al., 2019), allowing pathogens to infect and adapt to another host more effectively (Daugherty and Malik, 2012). For instance, a recent study that analyzed ACE2 receptors in Chinese horseshoe bats (*Rhinolophus sinicus*) found multiple such highly polymorphic sites in the receptor regions, which interacts with the spike proteins of a coronavirus isolated from the same species of bats named as SARSr-CoV (severe acute respiratory syndrome coronavirus isolated from *R. sinicus;* Guo et al., 2020). As expected, binding affinities of SARSr-CoV to these polymorphic receptors varied widely, making some cells more susceptible to viral entry than others. However, the most interesting aspect of their study was that, when tested upon human cell lines, some of these SARSr-CoV strains even showed higher binding affinity to human ACE2 compared to that of *R. sinicus*, hinting at their potential to cause spillover in overlapping human populations (Guo et al., 2020). Given the direct implications of these results in spillover and human health, we suggest the need for more such analyses to uncover the coevolutionary outcomes of pathogens from the diverse host interface (e.g., reservoirs vs. other host species), both at the spatial as well as the temporal scales.

Future studies can also test whether and how coevolving viral pathogens can maintain genetic polymorphism for adaptation to the host immune environment. Probing signatures of coevolution in accessory proteins that target host-associated restriction factors can aid understanding of the complex interplay between immune defense and viral adaptations. Indeed, the importance of such evolutionary processes has been implicated in previous studies where modified strains of HIV (simian tropic HIV-1 strain; stHIV-1) rapidly evolved to antagonize host restriction factor tetherin by acquiring mutations in the accessory protein Vpu within merely four passages through an atypical HIV-1 host species pigtailed macaques (*Maccaca nemestrina;* Hatziioannou et al., 2014). There are several other accessory genes as well such as Nef, Vif, and Vpr that have evolved in lentiviruses to counteract host antiviral immune responses (Sauter and Kirchhoff, 2018). Hence, in addition to finding links between host immune strategies (resistance vs. tolerance), life-history, and pathogen prevalence, revealing coevolutionary dynamics and resulting genetic diversification of circulating pathogens (i.e., key molecules involved in host entry, infectivity. and virulence) can greatly advance our understanding of their range expansion via spillover.

### Suggestion 4: set up controlled proof-of-principle laboratory coevolution studies to test hypotheses generated in the wild and provide mechanistic insights

It is important to note that due to the involvement of a multitude of factors ranging from genetics to environmental variations influencing animal populations, evaluating disease tolerance and pathogen spillover can be complicated in the wild. Data from field experiments can certainly provide information about larger patterns and processes such as heterogeneity in immune responses and genetic diversity in circulating pathogen strains, but creating a controlled empirical paradigm is perhaps necessary to generate more mechanistic insights into the actual micro-evolutionary processes. Finding greater pathogen diversity and prevalence in reservoir hosts with lower inflammatory responses, reduced rate of fitness loss, and increased polymorphism in pathogen receptor sites might indicate a potential correlation between coevolution, tolerance, and diverse zoonotic pathogen pool, but the causal link is still difficult to establish. Using common garden experimental setups that allow rearing and maintenance of well-characterized focal organisms under study in their semi-natural environmental conditions (e.g., large field enclosures for wild mice) can help us to partially overcome the uncertainties associated with quantifying parasite burden and estimating fitness traits in the wild (Barrett et al., 2019; Klemme et al., 2020).

**Figure 4.**
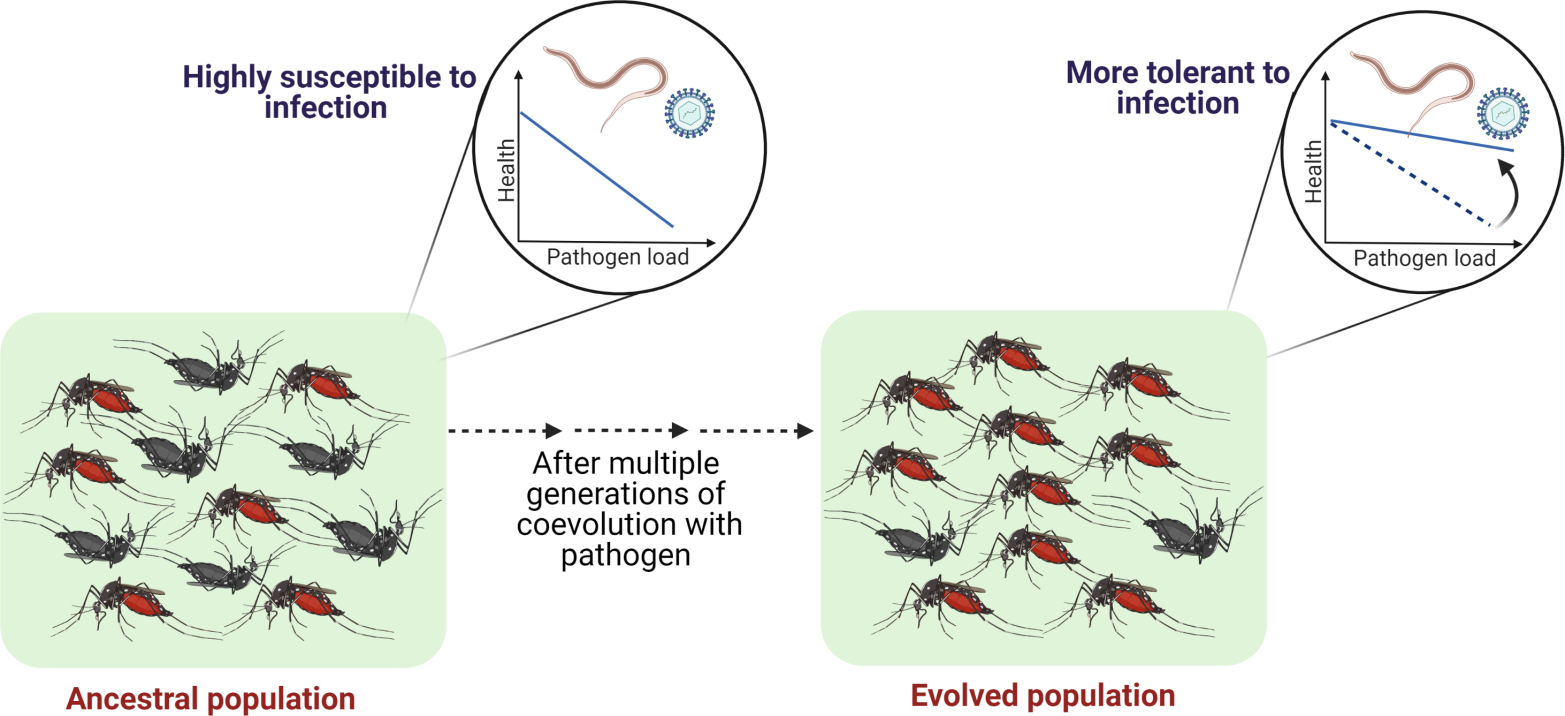
Outlining the controlled laboratory experimental evolution framework to test the link between coevolutionary history and tolerance. Susceptible host populations can be exposed to the coevolving pathogens at every generation and assayed periodically to estimate the changes in the tolerance level.

Yet it might be challenging to answer some of the most fundamental questions, such as do hosts actually evolve tolerance to their natural pathogens? If so, how do we track such evolutionary processes? Besides gathering clues from comparative studies using various host populations, laboratory experimental evolution using tractable animal models (with known biology and genomic information) can be an excellent alternative to test these possibilities (Khan et al., 2017b; Masri et al., 2013; Prasad and Joshi, 2003). They can enable us to directly track host-pathogen dynamics and test diverse hypotheses on the evolution of host tolerance, genetic diversifications of pathogens, and spillover risk to overlapping susceptible host populations. Owing to rapid generation time and easy maintenance, insect hosts, in particular, provide an excellent system to conduct such long-term evolution experiments (e.g., see Ford et al., 2020; Khan et al., 2017b; Mukherjee et al., 2019; but also see Kohl et al., 2016 for study in voles). While in principle any well-characterized insect model, with known biology and genetic information, can be used to test these basic hypotheses, mosquito hosts can be particularly useful both for the fundamental discovery as well as their direct relevance to human health (Huang et al., 2019). For example, filarial infections that exert strong selection pressure in mosquito hosts by inducing high mortality can be a valuable resource to test whether fitness costs are minimized by evolving tolerance (Aliota et al., 2010; Bartholomay, 2014). Experimental evolution studies can also be combined with a comparative dataset where multiple wild-caught mosquito populations are analyzed to quantify the natural variation in tolerance to filarial worms, followed by probing their underlying immune profiles. Subsequently, populations showing lower tolerance can be identified and subjected to repeated exposure to filarial infection across generations to test whether the level of tolerance can be further increased by modulating inflammatory responses. Such an integrated empirical framework might help in establishing the proposed causal link between coevolutionary history and pathogen tolerance (see Figure 4).

A similar experimental paradigm can also be used to test whether shared evolutionary history is responsible for tolerance in the vector hosts against their natural pathogens. For example, mosquito species *Armigeres subalbatus* is a natural vector for the zoonotic filarial worm *Brugia pahangi* whom they can tolerate, but not the morphologically and biologically similar pathogen *Brugia malayi* (Aliota et al., 2010; Aliota et al., 2007), which is perhaps not as prevalent as *B. pahangi* in the mosquito hosts (Muslim et al., 2013). In fact, mosquito hosts resist *B. malayi* infection using costly immune responses (Aliota et al., 2010; Aliota et al., 2007). Can long-term coevolution reverse such effects of *B. malayi* infection? By experimentally imposing long-term selection with the new pathogen *B. malayi*, we can verify the causal connection between the length of coevolutionary history and the level of host tolerance and parasite evolution. Subsequently, genetic analyses can uncover the mechanistic basis of adaptive changes in host immunity (e.g., possible modulation of costly inflammatory responses; Märkle et al., 2021) and pathogen replication and transmission potential (Siva-Jothy and Vale, 2021).

Laboratory evolution studies can also be implemented to track the evolutionary origin of established molecular mechanisms underlying tolerance strategies adopted by vector hosts. For example, both *A. albopictus* and *A. aegypti* can rapidly synthesize viral-derived DNA (vDNA), which is crucial for their tolerance and survival against chikungunya virus and dengue virus, respectively (Goic et al., 2016). How did such mechanisms evolve? A possible empirical framework is to (1) collect naturally isolated *Aedes* populations lacking (or with inherently lower) viral tolerance; (2) impose long-term viral selection to directly test whether stronger tolerance is correlated with increased vDNA synthesis; and (3) finally, test whether such evolved tolerance can be reversed by reducing vDNA synthesis (using reverse genetics) to verify its functional role (see Goic et al., 2016). Since previous experiments already demonstrated the role of tolerance in increasing the transmission intensity and vectorial capacity in mosquitoes (Dharmarajan et al., 2019), experiments showing direct evolution of parasite tolerance and infectivity in important vectors will make crucial contributions to public health (Lambrechts and Saleh, 2019).

## Conclusion and further implications for public health

In closing, as disease-causing pathogens from wild animals are emerging at an unprecedented rate across the globe, we must acknowledge that our understanding of specific ecological interactions and adaptive features of reservoir hosts is still at a nascent stage. A few theoretical models and experiments have provided broader insights into specific immune strategies to cater persistence of zoonotic pathogens (Alexander et al., 2012; Brook et al., 2020; White et al., 2018), but their oversimplistic assumptions might have limited inferential value in nature. To fill this gap, we have compiled a range of direct and indirect evidence of tolerance that can potentially explain the pathogen prevalence across host-pathogen systems, indicating its wider relevance to disease spread and spillover. However, despite the conceptual appeal, predictions based on the tolerance of reservoir hosts in catalyzing emerging infections still lack empirical rigor. There are no experiments that have thoroughly verified the whole process; that is, evolution of pathogen tolerance in reservoir animals to spillover. Recent analyses appear promising as they reveal the genetic basis differentiating the pathogen resistance from tolerance in critical human diseases such as HIV, where a high viral load often coincides with minimal disease progression as a feature of tolerance (Regoes et al., 2014). Also, there are experiments with bacteriophages that have confirmed that genetic variations are indeed required for viral emergence and host expansion (Dennehy et al., 2010). Still, targeted studies in natural reservoirs are needed to jointly probe the whole spectrum of tolerance to pathogen emergence, validating their cascading impacts on the spillover.

To this end, an integrated immune-centric understanding of naturally occurring variable infection outcomes across different host-pathogen systems and their specific ecological contexts, life-history, and evolutionary implications can be crucial. Systematic verification of the proposed links between pathogen prevalence, pathogen diversity, and host tolerance across a range of ecological contexts is needed, followed by deeper evolutionary insights into the maintenance of latent pathogen reservoirs and conditions that trigger spillover events. We believe that a hypothesis-driven experimental framework based on previous theoretical models is timely and will conceptually motivate a wide range of biologists to adopt a proactive disease surveillance program complemented with deeper ecological, evolutionary, and immunological thinking. Finally, we expect that our review will not only be relevant to the present crisis created by pandemic and emerging infections, but it will also provide a newer understanding of other important aspects of public health research such as infectious disease control (e.g., consequences of disease tolerance via vaccination) and the dynamics of noninfectious diseases (e.g., increased risk autoimmune disorders in geographical regions where improved hygiene has reduced pathogen burden; Bach, 2018).
